# Reliability of a new computerized equinometer based on Silfverskiöld test to measure gastrocnemius tightness

**DOI:** 10.1371/journal.pone.0284279

**Published:** 2023-04-12

**Authors:** Lucas Martinez, Matthieu Lalevée, Julien Beldame, Maxime L’Hermette, Helena Brunel, Franck Dujardin, Fabien Billuart

**Affiliations:** 1 Laboratoire d’Analyse du Mouvement, Institut de Formation en Masso-Kinésithérapie Saint Michel, Paris, France; 2 Unité de Recherche ERPHAN, UR 20201, Université de Versailles Saint Quentin, Versailles, France; 3 Service de chirurgie orthopédique et traumatologique, Centre Hospitalier Universitaire de Rouen, Rouen, France; 4 Clinique Mégival, Saint-Aubin-sur-Scie, France; 5 CETAPS, EA3832, Research Center for Sports and Athletic Activities Transformations, University of Rouen Normandy, Mont-Saint-Aignan, France; 6 UFR Simone Veil-Santé, avenue de la source de la Bièvre, Université de Paris-Saclay, Montigny-le-Bretonneux, France; King Khalid University, SAUDI ARABIA

## Abstract

**Background:**

Several tools exist to measure tightness of the gastrocnemius muscles; however, few of them are reliable enough to be used routinely in the clinic. The primary objective of this study was to evaluate the intra- and inter-rater reliability of a new equinometer. The secondary objective was to determine the load to apply on the plantar surface of the metatarsal heads to achieve the highest reliability when measuring gastrocnemius tightness.

**Methods:**

The equinometer consisted of a goniometer and an electronic dynamometer, hooked up to a computer. Three raters carried out three trials of passive dorsiflexion by applying controlled pressure to the metatarsal heads of both ankles in 29 healthy subjects under two experimental conditions: knee extended (KE) and knee flexed at 30 degrees (KF). The equinometer continuously recorded the ankle dorsiflexion values (in °) corresponding to each 1 N interval of plantar pressure between 4 N and 20 N. The intra- and inter-rater reliability of the ankle dorsiflexion were evaluated through the intra-class correlation (ICC) coefficients in each of the pressure intervals.

**Results:**

The intra-rater ICC in KE and KF was between 0.84 and 0.98. The inter-rater ICC in KE and KF was between 0.59 and 0.92. The pressure interval between 14 N and 15 N had the highest intra-rater (ICC = 1) and inter-rater reliability (0.87≤ICC≤0.99). A more refined analysis of this interval found that a load of 14.5 N yielded the best reliability.

**Conclusions:**

This compact equinometer has excellent intra-rater reliability and moderate to good inter-rater reliability. Since this reliability is optimal in the 14–15 N range, this load should be used going forward in clinical practice, especially when aiming to define a pathological threshold for tightness of the gastrocnemius muscles.

## Introduction

Various studies have shown that limited ankle dorsiflexion is a key contributor to decompensation of foot and ankle pathologies [1–3]. This limitation in ankle dorsiflexion can be caused by tightness of the gastrocnemius muscles [3–12]. The gastrocnemius tightness prevalence in the general population might be as high as 50% according to Kowalski et al. [13]. However, the current lack of a diagnostic threshold makes this number uncertain. This tightness is in most cases the result of a degenerative process, with a decrease in tendon elasticity over time [13, 14]. Gastrocnemius tightness, increasing with age, is known to be a predisposing factor for certain pathologies such as Achilles tendinopathy [3, 6, 7], plantar fasciitis [15], metatarsalgia [13, 14] or even hallux valgus [3, 5, 6]. These pathologies result in pain, reduced mobility, reduced participation in physical or social activities, thus affecting the quality of life of patients.

Given these factors, the diagnosis and treatment of gastrocnemius tightness appears to be crucial. However, it can be challenging to quantify this tightness. In clinical practice, there are several methods for evaluating gastrocnemius tightness. Currently, the Silfverskiöld test is widely used for this diagnosis [16, 17]. This test evaluates if the equinus contracture can be reduced by flexing the knee. However, there is no consensus on the amount of pressure to apply on the sole of the foot, nor on the increase in ankle dorsiflexion needed to make the diagnosis. Likewise, it is important to note that the reliability of this test is highly questionable. Indeed Molund et al. [17] have demonstrated a poor to average reliability for this test. A goniometer is also one of the methods used clinically. It is based on the practitioner’s end-of-stroke perception [18, 19] either with the subject weightbearing [20, 21] or with a measuring device [22, 23]. However, the main limitations of goniometer measurements are its lack of reliability, its operator-dependent nature and the fact that stiffness cannot be quantified solely by recording the change in an angle, without knowing the load applied to the foot [19].

To get around this lack of reliability and validity, several other methods have been proposed for evaluating ankle dorsiflexion. For example, Munteanu *et al*. [20] and Bennell *et al*. [21] proposed a functional approach that evaluates active ankle dorsiflexion during a controlled lunge motion. While this approach is reliable, it cannot be used in every clinical situation, especially when the patient is in pain, and does not control how much load is applied on the sole of the foot. An alternative method consists of evaluating the mobility in ankle dorsiflexion by measuring the angle between the foot and lower leg on a photograph when a load is applied to the metatarsal heads [24]. Beyond the challenges of applying this technique in routine clinical practice, the inability to stabilize the foot and the impossibility of quantifying the pressure placed on the metatarsal heads are significant limitations of these systems.

Lastly, measurement systems with a computerized interface have also been developed to measure the ankle range of motion with greater accuracy [8, 22, 23] but their cost and bulk means they are not well suited to clinical practice. Other than being affordable and easy to use clinically (transport, installation, use), a device to measure ankle dorsiflexion or “equinometer” must have good inter- and intra-rater reliability for measuring ankle dorsiflexion, while quantifying the load applied on the sole of the foot.

Given the limitations of the previously mentioned devices, we designed an electronic equinometer with computerized interface consisting of a goniometer to measure the ankle dorsiflexion and a dynamometer to quantify the load applied to the metatarsal heads. The entire unit is light, low profile and easy to transport.

Thus, the primary objective of this study was to evaluate the intra- and inter-rater reliability of a new equinometer for measuring gastrocnemius tightness. The secondary objective was to determine the load to apply on the metatarsal heads to achieve the highest possible reliability. We hypothesized that the equinometer will be reliable between and within raters and that a specific load value can be identified that will ensure the best measurement reliability.

## Materials and methods

The IRB of the Hôtel Dieu in Paris, France approved this study protocol on February 18, 2021 (Ref. # IORG0009918). Every subject was given an information letter describing the study. Subjects who agreed to participate in the study provided their written consent. The subjects’ data were anonymized.

### Subjects

Twenty-nine healthy adult volunteers were enrolled in the study between March 1 and April 30, 2021 (Table 1). The only inclusion criterion was being greater than 18 years of age. The subjects were recruited through an ad placed at a kinesiology and massage therapy training institute (IFMK Saint Michel, Paris, France) and at a hospital (Clinique Mégival, Dieppe, France).

**Table 1:** Subjects’ characteristics.

Features	Subjects
	Mean ± SD
	(min-max)
Number	29
Age (years)	24.60 ± 3.29 (22–32)
Sex (Male (M)/Female (F))	17M/12F
Height (cm)	175.33 ± 5.47 (167–185)
Mass (kg)	72.60 ± 8.91 (61–90)
Body Mass Index (kg/m^2^)	23.54 ± 1.86 (20.07–25.54)

Mean ± SD = Mean and standard deviation; min = minimum value; max = maximum value.

The exclusion criteria were the presence of pain in the lower limbs due to a recent injury or an active disease that prevents the examination from being done, and any cognitive or psychiatric disorder that prevents the subject from providing informed consent.

### Materials

**Fig 1:**
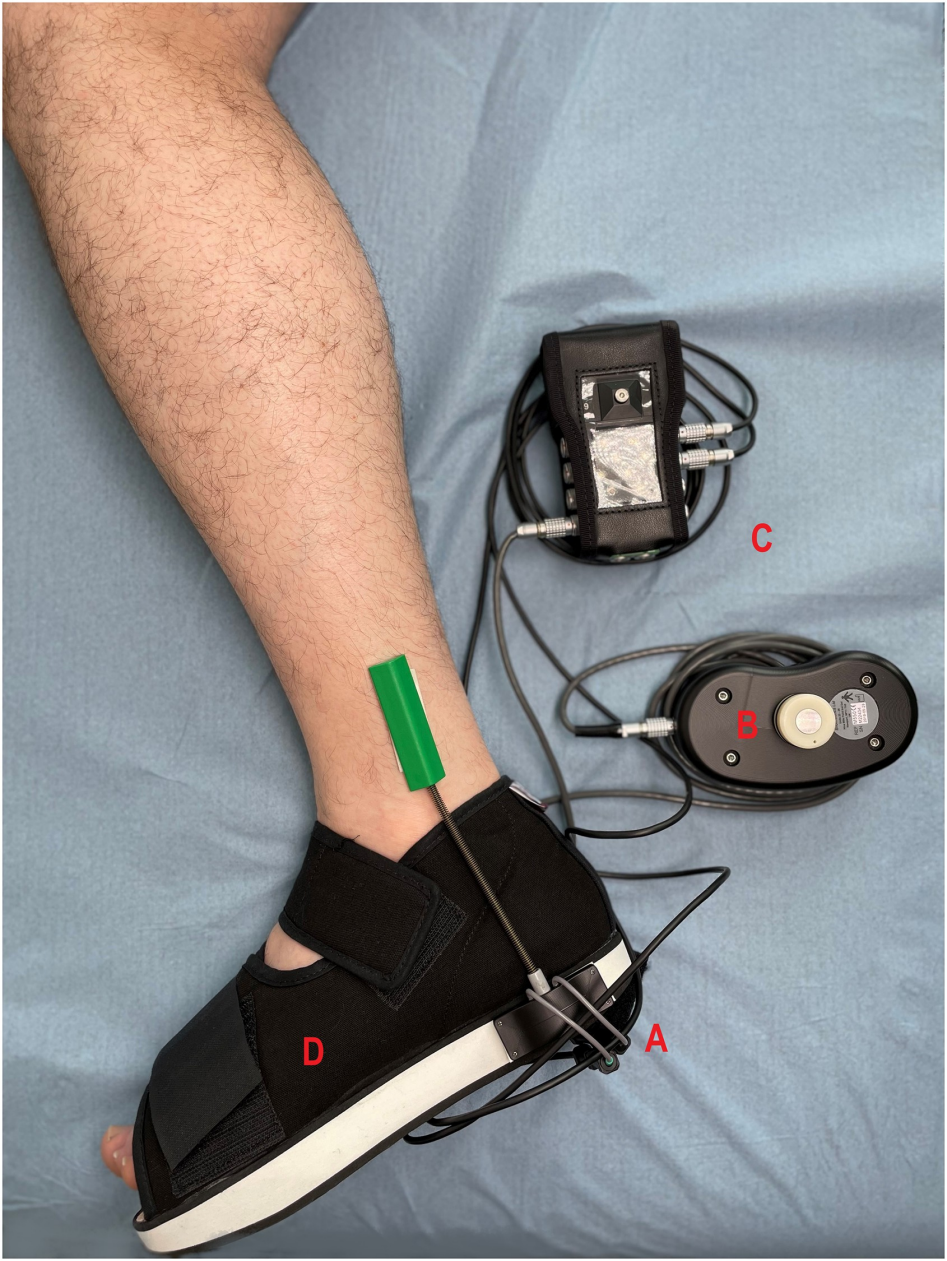
Equinometer with its various components: A = electronic twin-axis goniometer, B = MyoMeter electronic dynamometer, C = DataLOG and D = rigid shoe.

The equinometer (Fig 1) was designed to be easy to transport, easy to assemble and easy to use in a clinical context. The device that we developed to measure angles and load applied at the metatarsal heads had the following components:

- An electronic twin-axis goniometer (model SG110, Biometrics Ltd, Ladysmith, VA, USA)
- An electronic dynamometer (MyoMeter, Biometrics Ltd, Ladysmith, VA, USA)
- A portable data acquisition system (DataLOG, Biometrics Ltd, Ladysmith, VA, USA)
- A laptop equipped with software (Analysis Software, Biometrics Ltd, Ladysmith, VA, USA) for processing of data generated by the goniometer and dynamometer
- A rigid postoperative shoe (Perculight, Romans Industrie^™^, Romans sur Isère, France) in which the subject’s foot was positioned during testing

### Measurement protocol

The three raters (R1, R2, R3) were orthopedic surgeon and physiotherapists. They were all trained beforehand on their execution. The orthopedic surgeon was the rater with the longest experience using the equinometer.

#### Subject positioning

**Fig 2:**
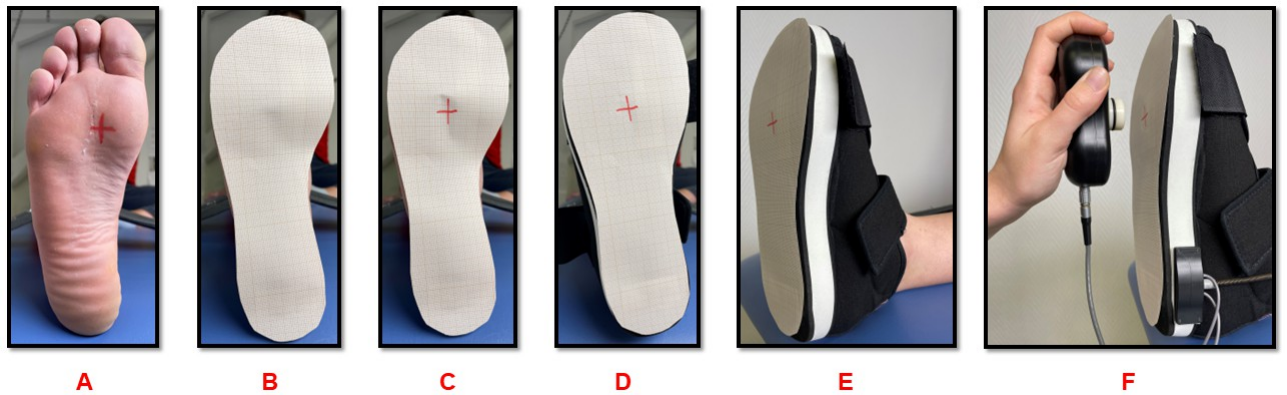
Identifying the head of the 2nd metatarsal. A: manual identification of the head of 2nd metatarsal; B: the adhesive graph paper is positioned on the sole of the foot; C: the marker of the head of the 2nd metatarsal head is transferred to the adhesive graph paper; D, E: the adhesive graph paper is attached to the shoe by checking the positioning of the foot in the shoe; F: the load is applied on the sole of the foot by the electronic dynamometer.

The subjects were installed supine on an adjustable-height table. Manually, and using a skin marker, the plantar surface of the head of the 2^nd^ metatarsal where the manual pressure will be applied to move the ankle into passive dorsiflexion was marked on the foot. This skin marker is transferred to an adhesive graph paper applied directly to the foot, and then reattached on the sole of the shoe (Fig 2).

Two experimental conditions were tested:

- Knee in full extension (KE)
- Knee in 30° flexion (KF). To hold the knee in 30° flexion [8], a half-roll was placed in the popliteal fossa. The size and position of this cushion was adjusted to obtain 30° flexion and was confirmed with the goniometer.

#### Subject set-up and goniometer calibration

**Fig 3:**
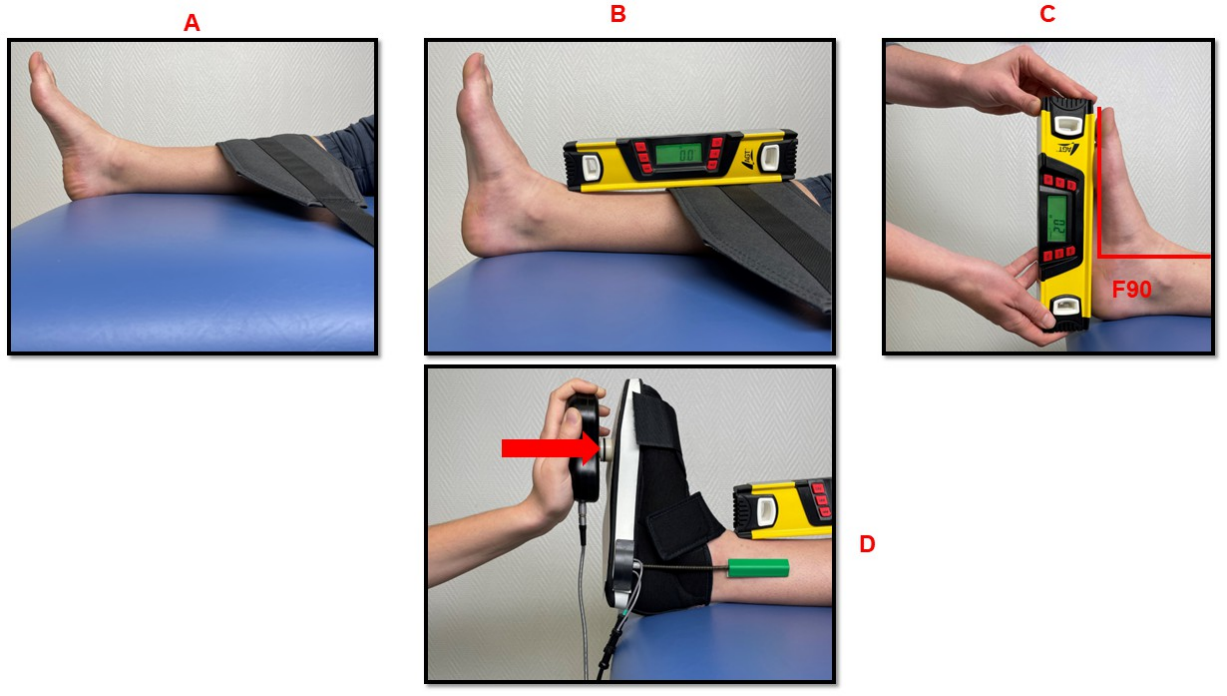
F90 measurement and positioning of the electronic goniometer on the sole of the rigid shoe and the medial malleolus. A: the leg segment is strapped to the table to keep it in position; B: the digital level is positioned on the tibial crest to ensure its horizontality; C: a second measurement is taken on the sole of the foot to ensure its verticality; D: one of the arms of the goniometer is placed on the sole of the shoe. The other arm is positioned on the medial malleolus, the application of force is perpendicular to the sole of the foot.

The subject was given a rigid postoperative shoe based on their regular shoe size. The goniometer was placed with double-sided tape on the sole of the shoe; one arm was placed on the shoe and the other on the medial malleolus (Fig 3). The ankle was placed in the anatomical reference position “F90°” (foot at 90° relative to the leg axis). This was used to calibrate the goniometer before measuring dorsiflexion. The “F90°” position was obtained with two measurements, using an external digital level (AGT^™^ Professional, Buggingen, Germany): a first measurement, with the digital level positioned on the tibial crest to ensure its horizontality (0°, Fig 3); then once this position is maintained, a second measurement is taken on the sole of the foot to ensure its verticality (0°, Fig 3). The angle formed between the sole of the foot and the leg segment is therefore 90° and constitutes the starting position for each measurement. The “F90°” is checked for each measurement following the same protocol. Once the “F90°” had been confirmed, the electronic goniometer was calibrated using the computer and software (set to zero).

#### Taking measurements

The rater stands across from the subject after having set the table height. The rater manually applies gradual pressure through the dynamometer on the sole of the shoe at the landmark previously made that corresponds to the head of the 2^nd^ metatarsal. The direction of the pressure was perpendicular to the sole. The dynamometer recorded the load applied by the rater on the subject’s foot. Three successive trials were done by the three raters on each of the subject’s two ankles. The trials were done with the lower leg under passive conditions; the subject had been instructed to let the rater move his/her ankle. The rater ensured there was no muscle contractions by looking at the tibialis anterior and extensor tendons subcutaneously. All the trials were done at 1-minute intervals, alternating between KE and KF:

- Trial 1 with the knee extended (KE)
  - One minute interval, new measurement of the F90°
- Trial 1 with the knee in 30° flexion (KF)
  - One minute interval, the rater removed the markers and then replaced them for Trial 2, new measurement of the F90°
- Trial 2 with the knee extended (KE)
  - One minute interval, new measurement of the F90°
- Trial 2 with the knee in 30° flexion (KF)
  - One minute interval, the rater removed the markers and then replaced them for Trial 3, new measurement of the F90°
- Trial 3 with the knee extended (KE)
  - One minute interval, new measurement of the F90°
- Trial 3 with the knee in 30° flexion (KF)
  - The rater removed the markers to position them on the other ankle

Six measurements were done on both ankles, resulting in 12 measurements per subject. The order in which the ankles (left or right) were tested was randomized for each subject. The 12 measurements of passive dorsiflexion were done by three different raters, one after the other, thus 36 measurements of passive dorsiflexion per subject. The markers were detached between trial 1 and 2 and also between trial 2 and 3. After each subject, markers were cleaned and then repositioned by each new rater with new double-sided tape.

### Statistical analysis

**Fig 4:**
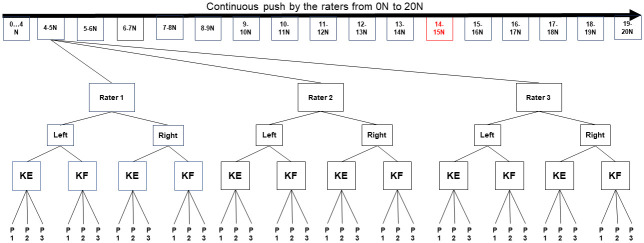
Example of how the data were collected between 4 N and 5 N. The DataLOG registers data continuously during the push phase between 0 and 20 N. Then the maximum angle value is extracted in the interval of interest. For example, for the interval between 4 N and 5 N, the data from each of the 3 raters are extracted for each leg (left and right) and in the two conditions (KE and KF). For each condition, three trials were done. The statical analysis was carried out for each condition. The procedure was repeated for each chosen interval, then a more refined analysis made in the interval between 14 N and 15 N, where the ICC values were the highest.

The DataLOG measured data continuously during the entire push and release phases by each rater in each trial. These data were then collected by the Analysis Software with output of load in Newtons and a corresponding angle in degrees (dependent variable). The data were then exported to a spreadsheet and processed by the study personnel. Data was extracted solely in the push phase on the sole of the shoe; 16 intervals of 1 N load were chosen for this study. The maximum value of the ankle dorsiflexion angle in degrees for each push phase was analyzed: between 4 N and 5 N, between 5 N and 6 N and so on until the interval between 19 N and 20 N. The statistical analysis was done using the software R^™^ (version 4.0.4, Bell Laboratories, Murray Hill, USA). Fig 4 summarizes how the data were analyzed.

The mean, SD and standard error of measurement (SEM) were calculated. The SEM was calculated as SEM = SD × (1-*r*)^1/2^, where “*r*” is the coefficient of reliability. The 95% confidence interval of the minimum detectable change (MDC) was used to look for clinically relevant changes in the dorsiflexion peak. The MDC is the minimum change that is not due to a measurement error. The MDC was calculated as the product of the SEM, the *z* score for the 95% confidence level and $\sqrt{2}$.

The intra-rater reliability was measured with Intraclass Correlation Coefficients (ICC) both for single measures, both comparing the three trials and taking the average of the three trials. The intra-rater reliability for single measures (SM) was calculated using a two-way random, single score ICC (ICC_2,1_) whereas the intra-rater reliability for average measures (AVG3M) was calculated using a two-way random, average score ICC (ICC_2,3_) [12].

The inter-rater reliability was calculated for each force level using a two-way random, single score ICC (ICC_2,1_).

For interrater reliability:

- The Single Measure ICC is an index for the reliability of each single rater
- The AVeraGe ICC is the average of the three trials for each rater
- The reliability is assessed by comparing the three raters

For the intrarater reliability:

- The Single Measure ICC is an index for the reliability of each trial
- The AVeraGe ICC is the average of the three raters for each trial
- The reliability is assessed by comparing the trials

The statistical analysis was repeated for both experimental conditions: KE and KF.

The loading interval where the inter- and intra-rater ICC was the best was then refined in 0.1-N increments (Fig 4).

## Results

Of the 58 ankles tested in this study, six had incomplete data due to recording errors. The left ankle of Subject 7 had no data. For Subject 8, data from rater 3 are missing for the left ankle. For Subject 9, one of the three trials by rater 3 on the right ankle is missing. For Subject 18, the measurements for the KF and KE are missing for rater 1. For Subject 28, data for KF by rater 3 are missing for the right ankle. The data from these six ankles were excluded from the analysis. These data were missing due to a problem with the software at the beginning of the study which was corrected. We had no problems with the latest measurements.

Table 2 shows the intra-rater reliability for ankle dorsiflexion measured by the three raters in the two conditions (KE and KF). The ICC values were between 0.84 and 0.99 (corresponding to a good and excellent reliability respectively, according Koo *et al*. [25]). The lowest ICC values were in the early intervals with the lowest applied forces; the lowest ICC (0.84) was found in the 4–5 N interval for rater 2 in the KE condition. Conversely, the highest ICC values were in the intervals with higher applied forces, up to a certain point beyond which the reliability decreased. In fact, the 14–15 N interval had the ICC closest to 1 for each rater for KE and KF (ICC *single measure* KE = 0.97 for R1, 0.96 for R2 and 0.98 for R3; ICC *single measure* KF = 0.98 for R1, 0.99 for R2 and 0.96 for R3, Table 2). The ICC for the 14–15 N interval for each rater is shown in Fig 6.

**Table 2:** Intra-rater reliability of single measures (SM) versus the average of 3 measures (AVG3M), ankle range of motions (degrees), MDC and SEM for ankle dorsiflexion measured by the 3 raters, with the knee extended (KF) and knee flexed (KF).

			4–5 N	5–6 N	6–7 N	7–8 N	8–9 N	9–10 N	10–11 N	11–12 N	12–13 N	13–14 N	14–15 N	15–16 N	16–17 N	17–18 N	18–19 N	19–20 N
**KE**	**R1**	SM (ICC_2,1_)	0.9 (0.86; 0.93)	0.91 (0.87; 0.94)	0.94 (0.91; 0.96)	0.94 (0.92; 0.96)	0.95 (0.93; 0.97)	0.95 (0.93; 0.97)	0.95 (0.93; 0.97)	0.96 (0.94; 0.97)	0.96 (0.94; 0.97)	0.96 (0.95; 0.98)	0.97 (0.96; 0.98)	0.97 (0.96; 0.98)	0.97 (0.96; 0.98)	0.97 (0.96; 0.98)	0.97 (0.96; 0.98)	0.97 (0.96; 0.98)
		AVG3M (ICC_2,3_)	0.96 (0.95; 0.98)	0.97 (0.95; 0.98)	0.98 (0.97; 0.99)	0.98 (0.97; 0.99)	0.98 (0.97; 0.99)	0.98 (0.98; 0.99)	0.98 (0.98; 0.99)	0.99 (0.98; 0.99)	0.99 (0.98; 0.99)	0.99 (0.98; 0.99)	0.99 (0.99; 0.99)	0.99 (0.99; 0.99)	0.99 (0.99; 0.99)	0.99 (0.99; 0.99)	0.99 (0.99; 0.99)	0.99 (0.99; 0.99)
		Mean ± SD (°)	9.7 ± 5.44	11.85 ± 6.15	13.86 ± 6.54	15.73 ± 6.91	17.42 ± 7.2	18.89 ± 7.4	20.26 ± 7.63	21.51 ± 7.85	22.63 ± 8.02	23.75 ± 8.25	24.35 ± 8.24	24.85 ± 8.44	25.51 ± 8.77	25.97 ± 8.88	26.35 ± 9.04	26.95 ± 9.3
		SEM (°)	0.43	0.49	0.52	0.55	0.57	0.59	0.61	0.62	0.64	0.66	0.67	0.7	0.75	0.76	0.81	0.85
		MDC (°)	1.2	1.35	1.44	1.52	1.58	1.63	1.68	1.73	1.76	1.83	1.86	1.95	2.07	2.12	2.23	2.35
	**R2**	SM (ICC_2,1_)	0.84 (0.78; 0.89)	0.86 (0.81; 0.91)	0.88 (0.83; 0.92)	0.9 (0.85; 0.93)	0.92 (0.88; 0.94)	0.94 (0.91; 0.96)	0.95 (0.93; 0.97)	0.95 (0.93; 0.97)	0.96 (0.95; 0.97)	0.96 (0.95; 0.98)	0.96 (0.95; 0.98)	0.96 (0.94; 0.97)	0.95 (0.93; 0.97)	0.96 (0.93; 0.97)	0.96 (0.94; 0.98)	0.96 (0.94; 0.98)
		AVG3M (ICC_2,3_)	0.94 (0.91; 0.96)	0.95 (0.93; 0.97)	0.96 (0.94; 0.97)	0.96 (0.95; 0.98)	0.97 (0.96; 0.98)	0.98 (0.97; 0.99)	0.98 (0.97; 0.99)	0.98 (0.98; 0.99)	0.99 (0.98; 0.99)	0.99 (0.98; 0.99)	0.99 (0.98; 0.99)	0.99 (0.98; 0.99)	0.98 (0.98; 0.99)	0.99 (0.98; 0.99)	0.99 (0.98; 0.99)	0.99 (0.98; 0.99)
		Mean ± SD (°)	9.65 ± 5.06	12.05 ± 5.39	14.16 ± 5.78	15.95 ± 6.24	17.58 ± 6.57	19.14 ± 6.82	20.53 ± 7.09	21.75 ± 7.33	22.83 ± 7.55	23.7 ± 7.85	24.47 ± 8.06	25.26 ± 8.25	25.49 ± 8.29	26.12 ± 8.49	26.89 ± 8.05	27.56 ± 8.3
		SEM (°)	0.4	0.43	0.46	0.5	0.52	0.54	0.56	0.58	0.6	0.63	0.66	0.69	0.71	0.74	0.72	0.75
		MDC (°)	1.11	1.18	1.27	1.37	1.44	1.5	1.56	1.61	1.66	1.76	1.84	1.91	1.98	2.05	1.99	2.08
	**R3**	SM (ICC_2,1_)	0.95 (0.93; 0.97)	0.96 (0.95; 0.98)	0.97 (0.95; 0.98)	0.97 (0.96; 0.98)	0.97 (0.95; 0.98)	0.96 (0.95; 0.98)	0.97 (0.95; 0.98)	0.97 (0.96; 0.98)	0.97 (0.96; 0.98)	0.98 (0.97; 0.98)	0.98 (0.97; 0.99)	0.98 (0.98; 0.99)	0.99 (0.98; 0.99)	0.99 (0.98; 0.99)	0.99 (0.98; 0.99)	0.99 (0.98; 0.99)
		AVG3M (ICC_2,3_)	0.98 (0.98; 0.99)	0.99 (0.98; 0.99)	0.99 (0.98; 0.99)	0.99 (0.99; 0.99)	0.99 (0.98; 0.99)	0.99 (0.98; 0.99)	0.99 (0.98; 0.99)	0.99 (0.99; 0.99)	0.99 (0.99; 0.99)	0.99 (0.99; 0.99)	0.99 (0.99; 1)	0.99 (0.99; 1)	1 (0.99; 1)	1 (0.99; 1)	1 (0.99; 1)	1 (0.99; 1)
		Mean ± SD (°)	11.98 ± 7.31	14.39 ± 7.57	16.34 ± 7.93	18.06 ± 8.19	19.54 ± 8.37	20.87 ± 8.57	21.76 ± 8.5	22.89 ± 8.72	23.92 ± 8.91	24.89 ± 9.07	25.81 ± 9.18	26.68 ± 9.3	27.48 ± 9.47	27.86 ± 9.63	28.32 ± 9.8	29.01 ± 10.13
		SEM (°)	0.59	0.61	0.63	0.66	0.68	0.69	0.69	0.71	0.73	0.74	0.75	0.76	0.77	0.8	0.84	0.88
		MDC (°)	1.62	1.68	1.76	1.82	1.88	1.92	1.92	1.97	2.02	2.05	2.08	2.11	2.14	2.22	2.34	2.44
**KF**	**R1**	SM (ICC_2,1_)	0.96 (0.94; 0.97)	0.96 (0.95; 0.98)	0.97 (0.96; 0.98)	0.97 (0.96; 0.98)	0.97 (0.96; 0.98)	0.97 (0.96; 0.98)	0.97 (0.96; 0.98)	0.97 (0.96; 0.98)	0.98 (0.96; 0.98)	0.98 (0.97; 0.99)	0.98 (0.97; 0.99)	0.98 (0.98; 0.99)	0.99 (0.98; 0.99)	0.99 (0.98; 0.99)	0.99 (0.99; 0.99)	0.99 (0.99; 1)
		AVG3M (ICC_2,3_)	0.99 (0.98; 0.99)	0.99 (0.98; 0.99)	0.99 (0.99; 0.99)	0.99 (0.99; 0.99)	0.99 (0.99; 0.99)	0.99 (0.98; 0.99)	0.99 (0.99; 0.99)	0.99 (0.99; 0.99)	0.99 (0.99; 0.99)	0.99 (0.99; 1)	0.99 (0.99; 1)	0.99 (0.99; 1)	1 (0.99; 1)	1 (0.99; 1)	1 (1; 1)	1 (1; 1)
		Mean ± SD (°)	18.14 ± 9.58	20.37 ± 10.15	22.22 ± 10.42	23.74 ± 10.78	24.44 ± 10.45	24.6 ± 10.22	25.37 ± 10.44	26.26 ± 10.62	27.06 ± 10.79	27.77 ± 10.96	28.46 ± 11.07	28.99 ± 11.33	29.48 ± 11.56	29.91 ± 11.96	30.29 ± 12.25	30.84 ± 12.32
		SEM (°)	0.77	0.81	0.83	0.85	0.84	0.84	0.87	0.88	0.9	0.91	0.92	0.95	0.98	1.05	1.1	1.15
		MDC (°)	2.13	2.25	2.29	2.37	2.32	2.34	2.41	2.45	2.49	2.53	2.56	2.65	2.73	2.92	3.06	3.2
	**R2**	SM (ICC_2,1_)	0.93 (0.9; 0.95)	0.95 (0.93; 0.97)	0.97 (0.96; 0.98)	0.98 (0.97; 0.98)	0.98 (0.97; 0.99)	0.98 (0.98; 0.99)	0.99 (0.98; 0.99)	0.99 (0.98; 0.99)	0.99 (0.98; 0.99)	0.99 (0.98; 0.99)	0.99 (0.98; 0.99)	0.99 (0.98; 0.99)	0.99 (0.98; 0.99)	0.99 (0.98; 0.99)	0.99 (0.98; 0.99)	0.99 (0.98; 0.99)
		AVG3M (ICC_2,3_)	0.98 (0.97; 0.98)	0.98 (0.98; 0.99)	0.99 (0.99; 0.99)	0.99 (0.99; 0.99)	0.99 (0.99; 1)	0.99 (0.99; 1)	1 (0.99; 1)	1 (0.99; 1)	1 (0.99; 1)	1 (0.99; 1)	1 (0.99; 1)	1 (0.99; 1)	1 (0.99; 1)	1 (0.99; 1)	1 (0.99; 1)	0.99 (0.99; 1)
		Mean ± SD (°)	17.37 ± 9.53	19.6 ± 10.14	21.48 ± 10.63	22.92 ± 11.03	24.17 ± 11.41	25.26 ± 11.78	26.28 ± 12.1	26.35 ± 11.98	27.1 ± 12.18	27.66 ± 12.68	28.26 ± 12.9	28.06 ± 12.63	28.12 ± 12.95	28.62 ± 13.16	29.26 ± 13.58	27.56 ± 13.2
		SEM (°)	0.76	0.8	0.84	0.87	0.9	0.93	0.96	0.98	0.99	1.07	1.09	1.09	1.15	1.17	1.24	1.35
		MDC (°)	2.1	2.23	2.34	2.42	2.51	2.59	2.66	2.71	2.76	2.96	3.01	3.01	3.2	3.25	3.43	3.73
	**R3**	SM (ICC_2,1_)	0.93 (0.89; 0.95)	0.95 (0.92; 0.96)	0.95 (0.92; 0.96)	0.95 (0.92; 0.96)	0.95 (0.93; 0.97)	0.96 (0.94; 0.97)	0.96 (0.94; 0.97)	0.96 (0.94; 0.97)	0.96 (0.94; 0.98)	0.96 (0.94; 0.97)	0.96 (0.94; 0.97)	0.96 (0.94; 0.97)	0.96 (0.94; 0.98)	0.96 (0.94; 0.97)	0.96 (0.94; 0.97)	0.97 (0.95; 0.98)
		AVG3M (ICC_2,3_)	0.97 (0.96; 0.98)	0.98 (0.97; 0.99)	0.98 (0.97; 0.99)	0.98 (0.97; 0.99)	0.98 (0.98; 0.99)	0.99 (0.98; 0.99)	0.99 (0.98; 0.99)	0.99 (0.98; 0.99)	0.99 (0.98; 0.99)	0.99 (0.98; 0.99)	0.99 (0.98; 0.99)	0.99 (0.98; 0.99)	0.99 (0.98; 0.99)	0.99 (0.98; 0.99)	0.99 (0.98; 0.99)	0.99 (0.98; 0.99)
		Mean ± SD (°)	18.01 ± 8.33	20.41 ± 8.64	22.2 ± 8.94	23.73 ± 9.2	24.99 ± 9.42	26.07 ± 9.66	27.02 ± 9.88	27.9 ± 10.16	28.61 ± 10.42	29.33 ± 10.56	29.42 ± 10.43	30.03 ± 10.53	30.17 ± 10.56	30.36 ± 10.77	31.19 ± 11	31.46 ± 11.13
		SEM (°)	0.67	0.69	0.72	0.74	0.75	0.77	0.79	0.82	0.85	0.86	0.87	0.88	0.9	0.94	0.98	1.03
		MDC (°)	1.85	1.92	1.98	2.04	2.09	2.14	2.19	2.28	2.36	2.39	2.41	2.43	2.49	2.6	2.72	2.85

KE = Knee Extended; KF = Knee Flexed; SM = Single Measures; AVG3M = Average of 3 measures; R1 = Rater 1; R2 = Rater 2; R3 = Rater 3. Mean ± SD = Mean and Standard Deviation of ankle Range Of Motion (Degrees); SEM = Standard Error of the Measurement.; MDC = Minimum Detectable Change.

**Fig 5:**
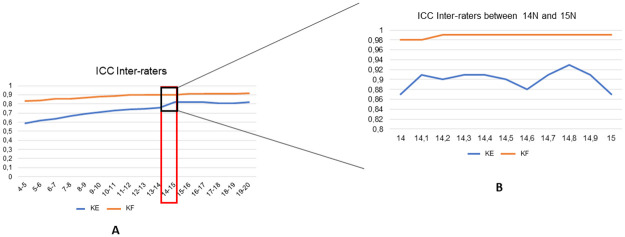
The inter-rater ICC for each interval in the KE and KF condition, with emphasis on the 14–15 N interval. A = inter-rater ICC for each interval in the two conditions. B = Emphasis on the interval between 14 N and 15 N that had the best inter-rater ICC. KE = knee extended, KF = knee flexed, X-axis: load applied in Newtons, Y axis: ICC values.

Table 3 shows the mean values with SD, the ICC with confidence interval, the SEM, the MDC of the ankle motion during KE and KF for the inter-rater assessment. The ICC where between 0.59 and 0.91 (corresponding to a moderate and excellent reliability respectively, according Koo *et al*. [25]) The ICC showed low agreement between the raters, especially for the lower force levels, with the lowest ICC (0.59) found in the 4–5 N interval in the KE condition. The ICCs were higher in the KF condition and followed the same trend as in the KE condition. The 14–15 N interval had the highest ICC both in the KE (ICC = 0.82; 95%CI = 0.74, 0.88) and KF (ICC = 0.9; 95%CI = 0.86, 0.94) conditions. Fig 5 shows the inter-rater ICC in 0.1 N increments between 14–15 N.

**Table 3:** Inter-rater reliability of ankle range of motion (degrees) with the knee extended (KF) and knee flexed (KF).

		4–5	5–6	6–7	7–8	8–9	9–10	10–11	11–12	12–13	13–14	14–15	15–16	16–17	17–18	18–19	19–20
KE	Mean ± SD	10.43 ± 5.93	12.76 ± 6.36	14.78 ± 6.75	16.57 ± 7.09	18.16 ± 7.34	19.62 ± 7.55	20.83 ± 7.66	22.04 ± 7.89	23.11 ± 8.09	24.11 ± 8.33	24.88 ± 8.45	25.61 ± 8.64	26.2 ± 8.85	26.68 ± 8.99	27.21 ± 8.98	27.87 ± 9.26
	ICC_2,1_ (95% CI)	0.59 (0.46; 0.7)	0.62 (0.5; 0.72)	0.64 (0.53; 0.74)	0.67 (0.56; 0.76)	0.69 (0.59; 0.78)	0.71 (0.61; 0.79)	0.73 (0.63; 0.81)	0.74 (0.65; 0.82)	0.75 (0.66; 0.83)	0.76 (0.67; 0.84)	0.82 (0.74; 0.88)	0.82 (0.75; 0.88)	0.82 (0.74; 0.88)	0.81 (0.73; 0.88)	0.81 (0.71; 0.88)	0.82 (0.73; 0.89)
	SEM (°)	0.47	0.51	0.54	0.56	0.59	0.6	0.61	0.63	0.65	0.67	0.69	0.71	0.75	0.77	0.79	0.83
	MDC (°)	1.31	1.4	1.49	1.56	1.62	1.67	1.7	1.75	1.8	1.87	1.92	1.98	2.07	2.13	2.19	2.3
KF	Mean ± SD (°)	17.83 ± 8.99	20.12 ± 9.53	21.97 ± 9.91	23.46 ± 10.27	24.54 ± 10.36	25.32 ± 10.53	26.24 ± 10.79	26.85 ± 10.88	27.6 ± 11.09	28.27 ± 11.35	28.71 ± 11.43	29.05 ± 11.45	29.29 ± 11.64	29.64 ± 11.93	30.26 ± 12.25	30.1 ± 12.22
	ICC_2,1_ (95% CI)	0.83 (0.76; 0.88)	0.84 (0.78; 0.89)	0.86 (0.8; 0.9)	0.86 (0.81; 0.91)	0.87 (0.82; 0.91)	0.88 (0.83; 0.92)	0.89 (0.84; 0.92)	0.9 (0.85; 0.93)	0.9 (0.85; 0.93)	0.9 (0.86; 0.94)	0.9 (0.86; 0.94)	0.91 (0.86; 0.94)	0.91 (0.86; 0.94)	0.91 (0.86; 0.94)	0.91 (0.86; 0.95)	0.92 (0.87; 0.96)
	SEM (°)	0.72	0.76	0.79	0.82	0.83	0.85	0.87	0.89	0.91	0.94	0.96	0.97	1.01	1.05	1.1	1.17
	MDC (°)	1.99	2.11	2.19	2.26	2.29	2.35	2.42	2.47	2.53	2.61	2.65	2.68	2.79	2.91	3.06	3.24

KE = knee extended; KF = knee flexed; Mean ± SD = Mean and Standard Deviation; ICC = Intraclass Correlation Coefficient; (95% CI) = 95% Confidence Interval; SEM = Standard Error of the Measurement.; MDC = Minimum Detectable Change; ° = degrees.

Tables 4 and 5 show the results of the refined analysis for the interval between 14 N and 15 N. Except for R2 in the KE condition and R3 in the KF condition, the intra-rater ICCs were all 1. For the inter-rater ICC, the highest values (>0.98, Table 4) were achieved when the testing was done in KF. An applied load of 14.5 N had the highest inter-rater and intra-rater ICC values.

**Table 4:** Intra-rater reliability of single measures (SM) versus the average of 3 measures (AVG3M) and 95% confidence intervals for ankle dorsiflexion measured by the 3 raters for the interval 14N-15N.

			14	14.1	14.2	14.3	14.4	14.5	14.6	14.7	14.8	14.9	15
KE	SM (ICC_2,1_)	R1	1 (1; 1)	1 (1; 1)	1 (1; 1)	1 (1; 1)	1 (1; 1)	1 (1; 1)	1 (1; 1)	1 (1; 1)	1 (1; 1)	1 (1; 1)	1 (1; 1)
		R2	0.99 (0.99; 1)	0.99 (0.99; 1)	0.99 (0.99; 1)	0.99 (0.99; 1)	0.99 (0.99; 0.99)	1 (1; 1)	0.99 (0.99; 1)	0.99 (0.99; 1)	0.99 (0.99; 1)	0.99 (0.99; 1)	0.99 (0.99; 0.99)
		R3	1 (1; 1)	1 (1; 1)	1 (1; 1)	1 (1; 1)	1 (1; 1)	1 (1; 1)	1 (1; 1)	1 (1; 1)	1 (1; 1)	1 (1; 1)	1 (1; 1)
	AVG3M (ICC_2,3_)	R1	1 (1; 1)	1 (1; 1)	1 (1; 1)	1 (1; 1)	1 (1; 1)	1 (1; 1)	1 (1; 1)	1 (1; 1)	1 (1; 1)	1 (1; 1)	1 (1; 1)
		R2	1 (1; 1)	1 (1; 1)	1 (1; 1)	1 (1; 1)	1 (1; 1)	1 (1; 1)	1 (1; 1)	1 (1; 1)	1 (1; 1)	1 (1; 1)	1 (1; 1)
		R3	1 (1; 1)	1 (1; 1)	1 (1; 1)	1 (1; 1)	1 (1; 1)	1 (1; 1)	1 (1; 1)	1 (1; 1)	1 (1; 1)	1 (1; 1)	1 (1; 1)
KF	SM (ICC_2,1_)	R1	1 (1; 1)	1 (1; 1)	1 (1; 1)	1 (1; 1)	1 (1; 1)	1 (1; 1)	1 (1; 1)	1 (1; 1)	1 (1; 1)	1 (1; 1)	1 (1; 1)
		R2	1 (1; 1)	1 (1; 1)	1 (1; 1)	1 (1; 1)	1 (1; 1)	1 (1; 1)	1 (1; 1)	1 (1; 1)	1 (1; 1)	1 (1; 1)	1 (1; 1)
		R3	0.99 (0.99; 0.99)	1 (1; 1)	0.99 (0.99; 1)	0.99 (0.99; 1)	1 (1; 1)	0.99 (0.99; 1)	0.99 (0.99; 1)	1 (1; 1)	0.99 (0.99; 1)	0.99 (0.99; 0.99)	1 (1; 1)
	AVG3M (ICC_2,3_)	R1	1 (1; 1)	1 (1; 1)	1 (1; 1)	1 (1; 1)	1 (1; 1)	1 (1; 1)	1 (1; 1)	1 (1; 1)	1 (1; 1)	1 (1; 1)	1 (1; 1)
		R2	1 (1; 1)	1 (1; 1)	1 (1; 1)	1 (1; 1)	1 (1; 1)	1 (1; 1)	1 (1; 1)	1 (1; 1)	1 (1; 1)	1 (1; 1)	1 (1; 1)
		R3	1 (1; 1)	1 (1; 1)	1 (1; 1)	1 (1; 1)	1 (1; 1)	1 (1; 1)	1 (1; 1)	1 (1; 1)	1 (1; 1)	1 (1; 1)	1 (1; 1)

KE = knee extended; KF = knee flexed; SM = Single Measures; AVG3M = Average of 3 measures; R1 = Rater 1; R2 = Rater 2; R3 = Rater 3.

**Table 5:** Inter-rater reliability of ankle range of motion (degrees) with the knee extended and knee flexed for the interval 14N-15N.

		14	14.1	14.2	14.3	14.4	14.5	14.6	14.7	14.8	14.9	15.0
**KE**	Mean ± SD	24.08±8.73	24.62±8.52	24.55±8.47	24.63±8.48	24.55±8.51	24.83±8.52	24.84±8.68	24.9±8.62	25.13 ± 8.56	25.13 ± 8.68	25.24 ± 8.56
	ICC_2,1_ (95% CI)	0.87 (0.82; 0.92)	0.91 (0.9; 0.94)	0.9 (0.84; 0.93)	0.91 (0.9; 0.94)	0.91 (0.91; 0.93)	0.9 (0.88; 0.93)	0.88 (0.87; 0.93)	0.91 (0.87; 0.93)	0.93 (0.86; 0.93)	0.91 (0.88; 0.93)	0.87 (0.87; 0.92)
	SEM	2.19	2.15	2.12	2.11	2.14	2.15	2.17	2.16	2.16	2.20	2.25
	MDC	6.06	5.97	5.89	5.86	5.93	5.96	6.02	5.99	6.0	6.1	6.2
**KF**	Mean ± SD	10.94±31.05	4.84±31.67	4.3±32.14	2.8±33.11	0.55±32.8	14.02±30.15	1.08±33.22	5.42±31.2	2.00 ± 31.99	1.45 ± 32.55	-1.78 ± 34.09
	ICC_2,1_ (95% CI)	0.98 (0.97; 0.99)	0.98 (0.97; 0.99)	0.99 (0.98; 0.99)	0.99 (0.98; 0.99)	0.99 (0.99; 1)	0.99 (0.99; 0.99)	0.99 (0.99; 1)	0.99 (0.98; 0.99)	0.99 (0.99; 0.99)	0.99 (0.98; 0.99)	0.99 (0.98; 0.99)
	SEM (°)	2.43	1.47	1.5	1.79	1.59	2.16	1.38	1.67	1.42	1.86	1.55
	MDC (°)	6.73	4.07	4.17	4.96	4.4	5.98	3.82	4.63	3.93	5.16	4.30

KE = knee extended; KF = knee flexed; Mean ± SD = Mean and Standard Deviation; ICC = Intraclass Correlation Coefficient; (95% CI) = 95% Confidence Interval; SEM = Standard Error of the Measurement.; MDC = Minimum Detectable Change;

## Discussion

**Fig 6:**
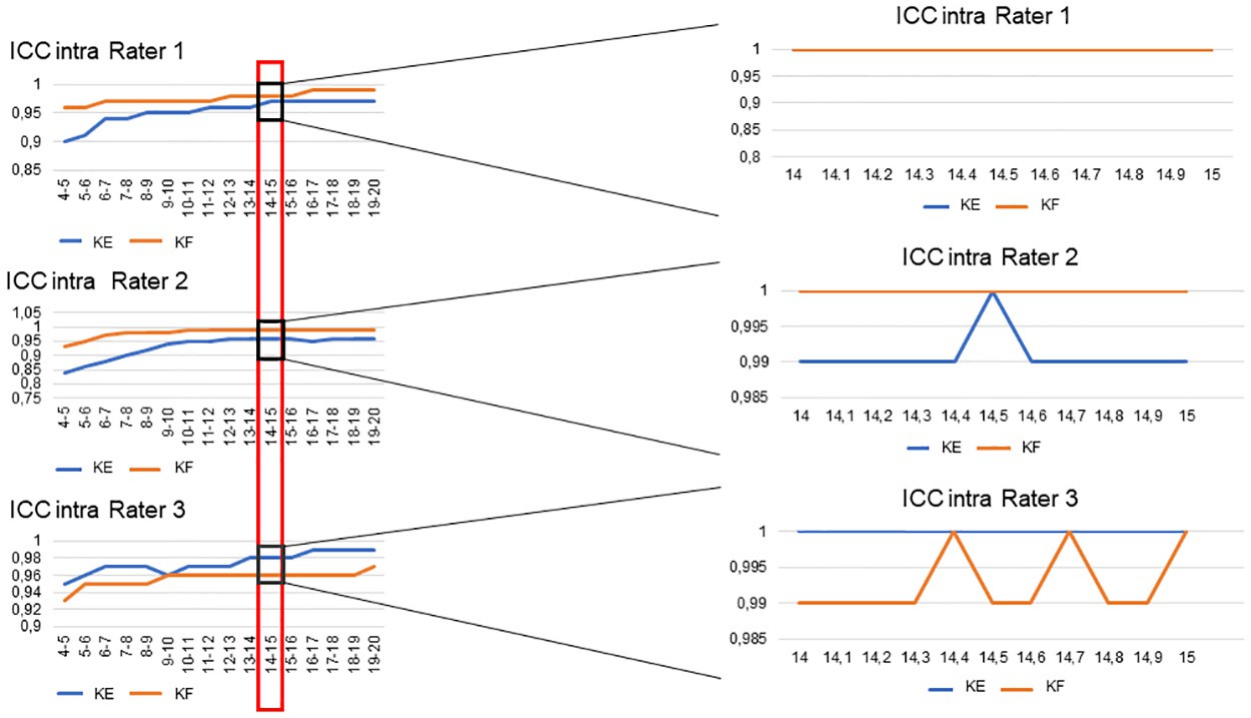
The intra-rater ICC for the three raters in the KE and KF condition, with emphasis on the 14–15 N interval. A = intra-rater ICC for each rater and each interval in the two conditions B = Emphasis on the interval between 14 N and 15 N that had the best intra-rater ICC. KE = knee extended, KF = knee flexed X-axis: load applied in Newtons, Y axis: ICC values.

The primary objective of this study was to evaluate the intra- and inter-rater reliability of a new equinometer based on Silfverskiöld test. Since the diagnosis of gastrocnemius tightness is made on the difference in dorsiflexion between the knee flexed (KF) and the knee extended (KE), we performed the measurements in the KE and KF positions. The secondary objective was to determine a load to apply on the metatarsal heads to ensure the best reliability. We hypothesized that the equinometer will be reliable in the two positions. Based on our reliability analysis, our hypothesis is confirmed. In fact, for the intra-rater reliability, the ICC values are all higher than 0.95, which translates to excellent reliability (Tables 2 and 4). For the inter-rater reliability, the ICC values are lower, pointing to moderate to good reliability between the three raters (Tables 3 and 5). However, there was a difference between the KE and KF conditions. The lowest ICC values were obtained in the KE condition at the lower end of the applied force range (Table 3). Conversely, the best inter-rater ICC values were obtained in the KF condition (Table 3). For the second objective–to determine a reliable load–the statistical analysis showed that the interval between 14–15 N had the best inter- and intra-rater reliability (Figs 5 and 6). In this interval, the intra-rater ICC in both conditions (KE, KF) was excellent (Table 4, Fig 6). The highest inter-rater reliability was always obtained with a flexed knee (ICC >0.98, Table 5, Fig 5). Within the 14–15 N interval, an applied force of 14.5 N appears to provide the best intra- and inter-rater reliability. However, it is important to note that this measurement is not a critical threshold determining gastrocnemius tightness.

Quantifying gastrocnemius tightness by measuring ankle dorsiflexion has a genuine clinical application [3]. Despite a confirmed need, the measurement is problematic due to a lack of affordable tools that can be used in routine clinical practice and with a well-defined protocol, especially the amount of force to apply when measuring the passive dorsiflexion. As suggested by Gatt *et al*. [10], quantifying the force applied to the foot when measuring dorsiflexion is a determining factor. Without this specific value, the evaluation has poor reliability because different practitioners are likely to use different loads during the procedure. Thus, it may be impossible to study the effectiveness of a treatment or to compare the results between different practitioners or different trials in the clinical or experimental context. The equinometer described here sought to solve this problem by providing a standardized method with a reliable load application. But the value presented as reliable in the present study does not constitute a threshold determining gastrocnemius tightness.

The intra-rater ICC obtained in this study was above 0.95, which is excellent reliability when compared to several studies of similar tools that were labelled as reliable [12, 26–28]. However, it should be noted that for Larsen *et al*. [26] and Worsley *et al*. [28] the investigations were carried out in weight bearing conditions. Finally, like Wilken et al. [12], in view of our results, it seems that a single measurement is sufficient to obtain excellent reliability, rather than 3. This element is important to consider in order to develop a tool that can be easily used in clinical routine. Our inter-rater ICC values are not as good as those reported by Wilken *et al*. [12], who however used pushing intervals higher than the present study (between 10 N.m and 25 N.m) in non-weightbearing condition (ICC > 0.92). However, according to Koo *et al*. [25] (where values less than 0.5 are indicative of poor reliability, between 0.5 and 0.75 are moderate reliability, between 0.75 and 0.9 are good reliability, and greater than 0.90 are excellent reliability), the inter-rater ICC values obtained in our study have good reliability for intervals above 12–13 N and moderate reliability for loads between 4 N and 12 N. The reliability was good to excellent in the 14–15 N interval [25]. The lower inter-rater reliability in the intervals other than 14–15 N can be explained by an accumulation of inaccuracies in certain steps of the measurement protocol inherent to the rater. The manual placement of the electronic goniometer remains operator-dependent and can lead to measurement variability if there are even (minimal) differences in placement between raters. Next, despite being standardized, the manual palpation of the load application point on the shoe via a foot landmark may have led to inaccuracies. Lastly, the orientation of the pushing force applied by the rater could affect the result. Wilken *et al*. believe that one of the reasons for the excellent inter-rater reliability in their study was that they followed closely a standardized protocol that reduced the risk of human error [12]. Meyer et *al*. [29], who also used pushing intervals higher than the present study (10 N.m) in non-weightbearing condition also found differences in the ICC between the KE and KF conditions. This difference can be attributed to the extensibility of the gastrocnemius muscles playing a role in the KE condition. In fact, the differences in extensibility between subjects could influence the measurement reliability, as evidenced by the confidence intervals of the ICC. Lastly, the MDC was between 1.3° and 2.3° in the KE condition and between 1.99° and 3.24° in the KF condition (Table 3). Also, the very low SEM (between 0.47° and 0.83° for KE and between 0.72° and 1.17° for KF, Table 3) is evidence of high measurement accuracy and minimal random or systematic errors. These values are consistent with other published values [26, 28, 30].

Determining the load application values to ensure the highest reliability was our study’s second objective. Our statistical analysis pointed to the 14–15 N interval, both for the intra- and inter-rater comparisons. It appears that a load of 14.5 N provided the highest reliability. To our knowledge, no other study has proposed such a precise load application interval to ensure the highest possible intra- and inter-rater reliability. Knowing the optimal force application value is relevant both clinically and experimentally. It is important to specify that the value presented as reliable in the present study does not constitute a threshold determining gastrocnemius tightness. Increasing the pressure would increase ankle dorsiflexion but would not change gastrocnemius tightness. The first step was to develop a reliable tool and the second step will be to define the angular threshold of dorsiflexion by comparing pathological and control patients. Once again, increasing load would only change the angular threshold but not the tightness. Also note that the SEM and MDC were the lowest within this interval (Tables 2 & 5). This is related to the fact that investigating such small force increments (0.1 N) within a 1 N interval likely increases the effect of any shaking or oscillating by the rater during the measurement. This may have gone unnoticed when looking at larger intervals.

Our study has several limitations. First, the reliability data were not obtained in ankles with documented pathology. As proposed by Worsley *et al*., [28] a more in-depth study in groups of patients with different conditions is needed to evaluate a tool under more realistic use conditions. Second, we did not validate our equinometer by comparing its output with other tools that are said to be reliable and valid. While some studies evaluated the validity of their tool with a motion analysis system [12], there is currently no gold standard. Third, the placement of the goniometer’s arms on the ankle then its fixation with double-side tape may have led to measurement inaccuracies, especially for force values within a given interval. We used specific anatomical landmarks to limit this effect. Also, the transposition of the load application point on the shoe from a point on the foot, even when using graph paper, may be a source of error. However, given our reliability results, this potential source of error could be considered acceptable. For the KF position, there is no standardization of knee flexion range in the literature, so we chose 30° of knee flexion according to DiGiovanni et al. [8] and because it facilitated the subject’s installation and the ability to maintain the knee in position. Lastly, our study has no direct clinical application. Its purpose was solely to validate a new equinometer that our team has developed. We only analyzed data between 4 and 20N, whereas some authors propose higher forces [10–12]. However, we designed our device according to Barouk *et al*. recommendations [5] in order to be as close as possible to the forces used during patients examination by physicians when performing a Silfverskiöld test. This step is an essential prerequisite to using this tool in clinical practice and for research purposes, with the goal of defining normal values and a pathological threshold value for gastrocnemius tightness.

## Conclusion

Our study presents a standardized method for measuring ankle dorsiflexion by using a new equinometer that combine digital goniometer measurement with quantification of the load applied to the foot. This method has good intra- and inter-rater reliability. The equinometer was most reliable when pushing on the foot with a force between 14 and 15N. However, this does not inform the angular threshold for diagnosing gastrocnemius tightness. This validates a new equinometer as a reliable device. Further studies should be performed using these parameters to attempt to define the pathological threshold for gastrocnemius tightness.
