# Supplementary material for: Reliability of a new computerized equinometer based on Silfverskiöld test to measure gastrocnemius tightness
Source: PLoS One. 2023 Apr 12;18(4):e0284279. doi: 10.1371/journal.pone.0284279 (PMC10096190; doi:10.1371/journal.pone.0284279)
Supplement: Peer review history

# Peer review: Decision Letter 0

**DOI:** 10.1371/journal.pone.0284279.r001

**Authors:** Danica Janicijevic

11 Oct 2022

PONE-D-22-19979Passive ankle dorsiflexion can be measured reliably using a new computerized equinometerPLOS ONE

Dear Dr. Lucas Martinez,

Thank you for submitting your manuscript to PLOS ONE. After careful consideration, we feel that it has merit but does not fully meet PLOS ONE’s publication criteria as it currently stands. Therefore, we invite you to submit a revised version of the manuscript that addresses the points raised during the review process.

==============================

ACADEMIC EDITOR:

Dear authors, the reviewers have sent their revisions asking for minor revisions. Specifically, reviewer one would like to see more reasoning about clinical relevance of your measurement, while second reviewer thinks that you need to add more clarifications in the method and results section. 

==============================

Please submit your revised manuscript by Nov 25 2022 11:59PM. If you will need more time than this to complete your revisions, please reply to this message or contact the journal office at plosone@plos.org. When you're ready to submit your revision, log on to https://www.editorialmanager.com/pone/ and select the 'Submissions Needing Revision' folder to locate your manuscript file.

Please include the following items when submitting your revised manuscript:

- A rebuttal letter that responds to each point raised by the academic editor and reviewer(s). You should upload this letter as a separate file labeled 'Response to Reviewers'.
- A marked-up copy of your manuscript that highlights changes made to the original version. You should upload this as a separate file labeled 'Revised Manuscript with Track Changes'.
- An unmarked version of your revised paper without tracked changes. You should upload this as a separate file labeled 'Manuscript'.

If you would like to make changes to your financial disclosure, please include your updated statement in your cover letter. Guidelines for resubmitting your figure files are available below the reviewer comments at the end of this letter.

If applicable, we recommend that you deposit your laboratory protocols in protocols.io to enhance the reproducibility of your results. Protocols.io assigns your protocol its own identifier (DOI) so that it can be cited independently in the future. For instructions see: https://journals.plos.org/plosone/s/submission-guidelines#loc-laboratory-protocols. Additionally, PLOS ONE offers an option for publishing peer-reviewed Lab Protocol articles, which describe protocols hosted on protocols.io. Read more information on sharing protocols at https://plos.org/protocols?utm_medium=editorial-email&utm_source=authorletters&utm_campaign=protocols.

We look forward to receiving your revised manuscript.

Kind regards,

Danica Janicijevic, Ph.D

Academic Editor

PLOS ONE

Journal Requirements:

When submitting your revision, we need you to address these additional requirements.

1. Please ensure that your manuscript meets PLOS ONE's style requirements, including those for file naming. The PLOS ONE style templates can be found at https://journals.plos.org/plosone/s/file?id=wjVg/PLOSOne_formatting_sample_main_body.pdf and 

https://journals.plos.org/plosone/s/file?id=ba62/PLOSOne_formatting_sample_title_authors_affiliations.pdf

2. We note that you have stated that you will provide repository information for your data at acceptance. Should your manuscript be accepted for publication, we will hold it until you provide the relevant accession numbers or DOIs necessary to access your data. If you wish to make changes to your Data Availability statement, please describe these changes in your cover letter and we will update your Data Availability statement to reflect the information you provide

Please review your reference list to ensure that it is complete and correct. If you have cited papers that have been retracted, please include the rationale for doing so in the manuscript text, or remove these references and replace them with relevant current references. Any changes to the reference list should be mentioned in the rebuttal letter that accompanies your revised manuscript. If you need to cite a retracted article, indicate the article’s retracted status in the References list and also include a citation and full reference for the retraction notice.

[Note: HTML markup is below. Please do not edit.]

Reviewers' comments:

Reviewer's Responses to Questions

**Comments to the Author**

1. Is the manuscript technically sound, and do the data support the conclusions?

The manuscript must describe a technically sound piece of scientific research with data that supports the conclusions. Experiments must have been conducted rigorously, with appropriate controls, replication, and sample sizes. The conclusions must be drawn appropriately based on the data presented. 

Reviewer #1: Yes

Reviewer #2: Partly

**********

2. Has the statistical analysis been performed appropriately and rigorously? 

Reviewer #1: Yes

Reviewer #2: Yes

**********

3. Have the authors made all data underlying the findings in their manuscript fully available?

The PLOS Data policy requires authors to make all data underlying the findings described in their manuscript fully available without restriction, with rare exception (please refer to the Data Availability Statement in the manuscript PDF file). The data should be provided as part of the manuscript or its supporting information, or deposited to a public repository. For example, in addition to summary statistics, the data points behind means, medians and variance measures should be available. If there are restrictions on publicly sharing data—e.g. participant privacy or use of data from a third party—those must be specified.

Reviewer #1: Yes

Reviewer #2: Yes

**********

4. Is the manuscript presented in an intelligible fashion and written in standard English?

PLOS ONE does not copyedit accepted manuscripts, so the language in submitted articles must be clear, correct, and unambiguous. Any typographical or grammatical errors should be corrected at revision, so please note any specific errors here.

Reviewer #1: Yes

Reviewer #2: Yes

**********

5. Review Comments to the Author

Please use the space provided to explain your answers to the questions above. You may also include additional comments for the author, including concerns about dual publication, research ethics, or publication ethics. (Please upload your review as an attachment if it exceeds 20,000 characters)

Reviewer #1: I appreciate your effort and your hard work to carry out this original study. I think that if you make the following clarifications, the article will be good for being published:

- I think that the introduction can be enlarged with more information about the pathology, for example, what ages it is more frequent to suffer tightness of the gastrocnemius muscles, or to highlight more the clinical importance of these measures.

From the methodological point of view, I would like you to explain better le following aspects:

- Why the knee is flexed at 30º?, There is some clinical test that is also carry out with those degrees of knee flexion or why do you select that patient position.

- Why do you use a external digital level to calibrate the starting position? I believe that it is not a very reliable measure, because we can have certain degrees of plantar flexion and the level marks zero, as it happens in the figure that you provide.

Reviewer #2: The authors have described a new tool for measuring passive ankle dorsiflexion. Accurate clinical measurement of ankle dorsiflexion is important for diagnosis and treatment of many lower limb pathologies and additional research into the area is welcome.

As per the plos guidelines, I believe this is original research and the findings have not been published previously. The paper is intelligible, meets ethical standards and adheres to appropriate reporting guidelines.

However, below are some comments, primarily in regard to the methods and interpretation of the results that I think should be addressed prior to publication. Many of the comments below are repetitive (sorry) as they refer to the recommended 14-15N force, which appears in the Abstract, Results and Discussion.

Abstract

I believe your conclusion needs to be changed as it is overstating what you have reported.

I do not think you have provided any evidence for the assertion in the conclusion that the 14-15 N range “…. should be used going forward in clinical practice, especially when aiming to define a pathological threshold for tightness of the gastrocnemius muscles.”

You have identified 14-15N as the most reliable of the ranges you have tested, however, you have not demonstrated in this study (or reported results from any other studies), that this level of force is sufficient to identify gastro tightness.

Introduction Section:

P3, Line 47 The references 3-7 are all father and son Barouk. Perhaps additional references from other eminent researchers in this field would be appropriate.

P3, Line 53 I agree the Silfverskiöld test is commonly used (and it is true there are limited other options), but I question that you can call it “gold” standard. Molund et al (ref 11) report ICC values for intra rater testing to be a poor 0.23, and as you note there is “no consensus on the amount of pressure to apply on the sole of the foot, nor on the increase in ankle dorsiflexion needed to make the diagnosis.” So really, not a good test at all.

P3 Line 58 Please correct the typo for the word “strock” as in “end-of-strock perception”.

The title of the paper refers to measuring “passive ankle dorsiflexion” but throughout the Abstract/Introduction/Conclusion you mostly refer to measuring gastrocnemius tightness rather than ankle dorsiflexion. I think it would be more accurate in light of what you have done in this trial for it to be changed to ankle dorsiflexion or calf tightness. If it is only gastrocnemius tightness that you wish to use this device for, then you need to explain why you are testing with the knee flexed.

Methods Section:

P6 Line 127 Could you please explain in more detail how the marker on the metatarsals, as per figure 2, is transferred to the bottom of the shoe, as obviously once the shoe is on the foot you cannot see the x marker.

P7 Line 136 Could you please reference why 30 degree flexion was used. I know it is to take away any effects of gastro, but use of particular values in the Methods requires referencing.

P8 Line 176 You state the markers were replaced by each tester. Please can you give more detail on how the testing was conducted. For instance

• did each rater put their own mark on the foot/shoe separately for each participant?

• Did each rater remove the marker after each trial. i.e. Did they conduct the first knee extended trial then the knee flexed trial, then remove the marker and replace it before the second knee flexed trial? Or did they conduct a trial for knee flexed and extended then go to the next participant?

I am looking for clarification that the markers were removed after each rater completed each trial.

Rather than the current Figure 2, a picture of the rater performing the procedure with the rater applying the force to the foot with the dynamometer would be more informative. Just to give the reader a clearer understanding of the testing setup.

Statistics Section:

P9 Line 188 You analysed data in 1N blocks from 4N to 20N. Please can explain your reasoning (with references) for choosing this level of force. This is a relatively low level of force compared to other studies you have quoted such as Wilken et al (2011) who applied 45 to 111N (5 to 25Nm) of force.

P9 Line 192 Please change ET to SD in the formula used for SEM, as you use SD in the rest of the document.

P9 Line 198 Please can you state which form of ICC you used i.e. ICC (3,1)

Also, in the discussion you cite Koo et al (ref 25) for the level of reliability (good, excellent etc). I think it would be helpful to the readers if you put the range of values for each level of reliability as per Koo in this section.

P9 Line 198 I am a bit confused by the single measures (SM) and average measures (AVG3M). I am not a statistics expert, but I suspect that if I am confused other readers will be too.

• Could you please describe why you are reporting single measures and average measures.

o I am guessing it is to show whether the test result is acceptable with just one measure (which makes it faster in clinic), or if performing it 3 times and using an average gives more accurate results. If that is the case, then you should make a comment in the results and discussion sections as to whether 1 or 3 trials is more acceptable and should be conducted when this device is used in the future.

• Can you clarify what you mean by single measures (SM) and average measures (AVG3M).

o I assume the single measure (SM) is the reliability across each of the three trials for each participant for each rater?

o And that the AVG3M, is the average of the 3 trials for each participant for each rater? If this is the case then I am unsure what other value you are comparing this to, to calculate reliability, because you did not do a second set of three trials to produce another average.

Results Section:

P10 Line 220 It would be useful to the reader if you stated the intra-rater results with the range of ICC and the level of reliability as per Koo et al.

P10 Line 220 It would be useful to the reader if you stated the inter-rater results with the range of ICC and the level of reliability as per Koo et al.

P15 Table 4 As per my earlier comment regarding SM and AVG3M, I am not clear on what this is being reporting.

In the discussion you comment on the low SEM and MDC for inter rater testing. However, you have not reported SEM or MDC or mean ROM for intra tester reliability. I understand why you have not done this as the table with all 3 raters would be enormous. Nonetheless, it would be useful and important so readers can decide whether 1 or 3 trials are required for accurate results. Is it possible to include a table (similar format to table 3 inter rater results) of perhaps just one of the raters individual results with mean ROM, SEM and MDC to give an indication of the possible values. Or maybe it could be supplementary data.

Discussion Section:

P18 Line 286 I agree with what you have written in this paragraph except for this sentence. The device is reliable for the levels of force applied but there has been no determination or evidence that your “optimal” force of 14-15N can identify gastrocnemius restriction.

P18, Line 288 This paragraph could do with more detail. Comparing and contrasting your results to other similar studies is important. But in this area the devices used are often quite different. So, a sentence on what device was used in the studies you have quoted, the amount of force applied and whether it was conducted weight bearing on non-weightbearing would be helpful.

P18, Paragraph from Line 311 Again the issue with calling 14 to 15N an “optimal” value. You have identified that it is optimal for producing the most reliable inter/intra rater results (and only within the limited range of forces you tested). You have not shown it to be optimal for identifying gastro tightness or any other pathology or indeed in what context that range is important. Consequently, I do not think you have supplied sufficient justification for recommending this range to be used for future testing.

Conclusion

P20 Line 343 As per my previous comments, I do not think you can say the optimal level is appropriate for identifying gastro tightness.

**********

6. PLOS authors have the option to publish the peer review history of their article (what does this mean?). If published, this will include your full peer review and any attached files.

If you choose “no”, your identity will remain anonymous but your review may still be made public.

**Do you want your identity to be public for this peer review?** For information about this choice, including consent withdrawal, please see our Privacy Policy.

Reviewer #1: No

Reviewer #2: No

**********

[NOTE: If reviewer comments were submitted as an attachment file, they will be attached to this email and accessible via the submission site. Please log into your account, locate the manuscript record, and check for the action link "View Attachments". If this link does not appear, there are no attachment files.]

While revising your submission, please upload your figure files to the Preflight Analysis and Conversion Engine (PACE) digital diagnostic tool, https://pacev2.apexcovantage.com/. PACE helps ensure that figures meet PLOS requirements. To use PACE, you must first register as a user. Registration is free. Then, login and navigate to the UPLOAD tab, where you will find detailed instructions on how to use the tool. If you encounter any issues or have any questions when using PACE, please email PLOS at figures@plos.org. Please note that Supporting Information files do not need this step.

# Author comment: Author response to Decision Letter 0

**DOI:** 10.1371/journal.pone.0284279.r002

18 Nov 2022

To the Editorial Board and the Reviewers,

We would like to thank the Editorial Board and the Reviewers for the precious comments on our manuscript. These comments will certainly make the manuscript more robust and understandable. We have considered the comments of the 2 reviewers and we have modified our manuscript accordingly. We hope that our modifications will meet your expectations. We left in this letter the comments from the 2 reviewers. As requested, you will find in our new submission, a version highlighting the changes made with comments visible in Word with elements highlighted in yellow (Revised Manuscript with tracked changes). You will also find an unmarked version without tracked changes. 

We await your decision and hope for a favorable outcome. 

Yours faithfully

The authors 

For Reviewer #1:

I appreciate your effort and your hard work to carry out this original study. I think that if you make the following clarifications, the article will be good for being published:

I think that the introduction can be enlarged with more information about the pathology, for example, what ages it is more frequent to suffer tightness of the gastrocnemius muscles, or to highlight more the clinical importance of these measures.

We have added elements in the introduction on the physiopathology, the target population of gastrocnemius tightness and its consequences.

P3-L48: “ The gastrocnemius tightness prevalence in the general population might be as high as 50% according to Kowalski et al. However, the current lack of a diagnostic threshold makes this number uncertain. This tightness is in most cases the result of a degenerative process, with a decrease in tendon elasticity over time [13,14]. Gastrocnemius tightness, increasing with age, is known to be a predisposing factor for certain pathologies such as Achilles tendinopathy [3,6,7], plantar fasciitis [15], metatarsalgia [13,14] or even hallux valgus [3,5,6]. These pathologies result in pain, reduced mobility, participation in physical or social activities, thus affecting the patients’ quality of life.”

From the methodological point of view, I would like you to explain better le following aspects:

- Why the knee is flexed at 30º?, There is some clinical test that is also carry out with those degrees of knee flexion or why do you select that patient position.

Thank you for this insightful comment. There is no standardization of knee flexion range in the literature. Some studies propose 90°, others 45° and some 20° (according to the review carried out by Gatt et al. [11]). However, we chose 30° for the following reasons and the related reference: DiGiovanni et al. [8] estimate the gastrocnemius to be totally relaxed above 25° of knee flexion. We have also chosen 30° because it facilitated the subject’s installation and the ability to maintain the knee in position.

P8 L152: “Knee in 30° flexion (KF). To hold the knee in 30° flexion [8], a half-roll was placed in the popliteal fossa”

P22 L 387: “For the KF position, there is no standardization of knee flexion range in the literature, so we chose 30° of knee flexion according to DiGiovanni et al. [8] because it facilitated the subject’s installation and the ability to maintain the knee in position.”

Why do you use a external digital level to calibrate the starting position? I believe that it is not a very reliable measure, because we can have certain degrees of plantar flexion and the level marks zero, as it happens in the figure that you provide.

Thank you for this remark. Indeed, the explanation of this measurement lacks precision as well as the Figure 3. In fact, we use two measurements with the digital level. The first measurement is presented in Figure 3 which ensures the horizontality of the leg segment. Once the horizontal position of the leg segment is locked, a second measurement is made on the sole of the foot to ensure its verticality, thus forming a 90° angle between the sole of the foot and the tibia, a position that we have called “F90°”, and constituting our starting position for each measurement in order to be the most reliable. Our explanations were not very clear but have been reformulated in the text. Similarly, we present a new Figure 3 including the two measurements.

P8 L160: “The “F90°” position was obtained with two measurements, using an external digital level (AGT™ Professional, Buggingen, Germany): a first measurement, with the digital level positioned on the tibial crest to ensure its horizontality (0°, Fig. 3); then once this position is maintained, a second measurement is taken on the sole of the foot to ensure its verticality (0°, Fig 3). The angle formed between the sole of the foot and the leg segment is therefore 90° and constitutes the starting position for each measurement. The “F90°” is checked for each measurement following the same protocol. Once the “F90°” had been confirmed, the electronic goniometer was calibrated using the computer and software (set to zero).”

See new Figure 3

For Reviewer #2:

The authors have described a new tool for measuring passive ankle dorsiflexion. Accurate clinical measurement of ankle dorsiflexion is important for diagnosis and treatment of many lower limb pathologies and additional research into the area is welcome.

As per the plos guidelines, I believe this is original research and the findings have not been published previously. The paper is intelligible, meets ethical standards and adheres to appropriate reporting guidelines.

However, below are some comments, primarily in regard to the methods and interpretation of the results that I think should be addressed prior to publication. Many of the comments below are repetitive (sorry) as they refer to the recommended 14-15N force, which appears in the Abstract, Results and Discussion.

Introduction Section:

P3, Line 47 The references 3-7 are all father and son Barouk. Perhaps additional references from other eminent researchers in this field would be appropriate.

Indeed, thank you for this comment. Barouk’s works are the major references on these questions but there are other eminent authors that we have not mentioned. We added them in this part. We have thus added the works of Gatt et al., Digiovanni et al., Wilken et al.

P3-L48: “This limitation in ankle dorsiflexion can be caused by tightness of the gastrocnemius muscles [3–12]”

P3, Line 53 I agree the Silfverskiöld test is commonly used (and it is true there are limited other options), but I question that you can call it “gold” standard. Molund et al (ref 11) report ICC values for intra rater testing to be a poor 0.23, and as you note there is “no consensus on the amount of pressure to apply on the sole of the foot, nor on the increase in ankle dorsiflexion needed to make the diagnosis.” So really, not a good test at all.

We totally agree, the Silfverskiold test is a very widespread test and is widely used in clinical routine, although ultimately its reliability is not good. The term "gold standard" that we wrote is overused. We meant that it is widely used. But actually “gold standard” is not the right term. we took the sentence and argued with the literature.

P3-L59: “Currently, the Silfverskiöld test is widely used for this diagnosis [16,17]”

P3- L63: “Likewise, it is important to note that the reliability of this test is highly questionable. Indeed Molund et al. [17] have demonstrated a poor to average reliability for this test”

P3 Line 58 Please correct the typo for the word “strock” as in “end-of-strock perception”.

We apologize for this typo. We modified it:

P3 L65-66: “It is based on the practitioner’s end-of-stroke perception”

The title of the paper refers to measuring “passive ankle dorsiflexion” but throughout the Abstract/Introduction/Conclusion you mostly refer to measuring gastrocnemius tightness rather than ankle dorsiflexion. I think it would be more accurate in light of what you have done in this trial for it to be changed to ankle dorsiflexion or calf tightness. If it is only gastrocnemius tightness that you wish to use this device for, then you need to explain why you are testing with the knee flexed.

Thank you for this remark. Indeed, it turns out that the title of the article is ultimately not very suitable and that it would be more relevant to mention “gastrocnemius tightness”. We have thus modified the title and the various passages which led to confusion in the article. In agreement with Barouk et al. [5] and Silfverskiold the diagnosis of gastrocnemius tightness is made on the difference in dorsiflexion between the knee flexed and the knee extended and it is therefore essential that the measurement be reliable in these two positions.

Title: “Reliability of a new computerized equinometer based on Silfverskiöld test to measure gastrocnemius tightness”

Abstract : “The primary objective of this study was to evaluate the intra- and inter-rater reliability of a new equinometer. The secondary objective was to determine the load to apply on the plantar surface of the metatarsal heads to achieve the highest reliability when measuring gastrocnemius tightness.”

P4-L91: “Thus, the primary objective of this study was to evaluate the intra- and inter-rater reliability of a new equinometer for measuring gastrocnemius tightness. The secondary objective was to determine the load to apply on the metatarsal heads to achieve the highest possible reliability. We hypothesized that the equinometer will be reliable between and within raters and that a specific load value can be identified that will ensure the best measurement reliability.”

P19-L295: “The primary objective of this study was to evaluate the intra- and inter-rater reliability of a new equinometer based on Silfverskiöld test. Since the diagnosis of gastrocnemius tightness is made on the difference in dorsiflexion between the knee flexed (KF) and the knee extended (KE), we performed the measurements in the KE and KF positions. The secondary objective was to determine a load to apply on the metatarsal heads to ensure the best reliability. We hypothesized that the equinometer will be reliable in the two positions.”

Methods Section:

P6 Line 127 Could you please explain in more detail how the marker on the metatarsals, as per figure 2, is transferred to the bottom of the shoe, as obviously once the shoe is on the foot you cannot see the x marker.

Thank you for this remark. Indeed, we have not sufficiently detailed the step for the transition from the foot to the shoe. Indeed, after having manually located and noted the head of the 2nd metarsal, this marking is transferred to adhesive graph paper applied to the foot, and then repositioned on the sole of the shoe. We have modified the sentence on page 7 and made a new Figure 2:

P7 L140: “Manually, and using a skin marker, the plantar surface of the head of the 2nd metatarsal where the manual pressure will be applied to move the ankle into passive dorsiflexion was marked on the foot. This skin marker is transferred to an adhesive graph paper applied directly to the foot, and then reattached on the sole of the shoe (Fig 2).”

See new Figure 2

P22 L384: “Also, the transposition of the load application point on the shoe from a point on the foot, even when using graph paper, may be a source of error. However, given our reliability results, this potential source of error could be considered acceptable.”

P7 Line 136 Could you please reference why 30 degree flexion was used. I know it is to take away any effects of gastro, but use of particular values in the Methods requires referencing.

Thank you for this insightful comment. There is no standardization of knee flexion range in the literature. Some studies propose amplitudes at 90°, others at 45° and some at 20° (according to the review carried out by Gatt et al. [11]). However, we chose 30° for the following reasons and the related reference: DiGiovanni et al. [8] estimate that the gastrocnenius are totally relaxed for flexion greater than 25°. Thus, we chose 30° of flexion in view of this reference and because it facilitated the installation of the subjects and the ability to maintain the knee in position.

P8 L152: “Knee in 30° flexion (KF). To hold the knee in 30° flexion [8], a half-roll was placed in the popliteal fossa”

P22 L 387: “For the KF position, there is no standardization of knee flexion range in the literature, so we chose 30° of knee flexion according to DiGiovanni et al. [8] because it facilitated the subject’s installation and the ability to maintain the knee in position.”

P8 Line 176 You state the markers were replaced by each tester. Please can you give more detail on how the testing was conducted. For instance

• did each rater put their own mark on the foot/shoe separately for each participant?

• Did each rater remove the marker after each trial. i.e. Did they conduct the first knee extended trial then the knee flexed trial, then remove the marker and replace it before the second knee flexed trial? Or did they conduct a trial for knee flexed and extended then go to the next participant?

I am looking for clarification that the markers were removed after each rater completed each trial.

Indeed, we were not clear on the way in which the measures were taken. Each rater placed the markers on a subject's ankle. He performs the KE measurement, then the KF measurement with one-minute intervals. He removes the markers and repeats the protocol 2 more times. Then he removes the markers, replaces them on the other leg and performs the same measurements in the same order. Finally, he removes the markers, cleans them. Rater 2 succeeds him without having seen the manipulation and repeats the same protocol and so on for the 3rd rater. The same protocol is then repeated on the following subjects.

We have modified the paragraph accordingly to your remarks, 

P9-L184: “All the trials were done at 1-minute intervals, alternating between KE and KF:

• Trial 1 with the knee extended (KE)

o One minute interval, new measurement of the F90° 

• Trial 1 with the knee in 30° flexion (KF)

o One minute interval, the rater removed the markers and then replaced them for Trial 2, new measurement of the F90°

• Trial 2 with the knee extended (KE)

o One minute interval, new measurement of the F90° 

• Trial 2 with the knee in 30° flexion (KF)

o One minute interval, the rater removed the markers and then replaced them for Trial 3, new measurement of the F90°

• Trial 3 with the knee extended (KE)

o One minute interval, new measurement of the F90° 

• Trial 3 with the knee in 30° flexion (KF)

o The rater removed the markers to position them on the other ankle

Six measurements were done on both ankles, resulting in 12 measurements per subject. The order in which the ankles (left or right) were tested was randomized for each subject. The 12 measurements of passive dorsiflexion were done by three different raters, one after the other, thus 36 measurements of passive dorsiflexion per subject. The markers were detached between trial 1 and 2 and also between trial 2 and 3. After each subject, markers were cleaned and then repositioned by each new rater with new double-sided tape.”

Rather than the current Figure 2, a picture of the rater performing the procedure with the rater applying the force to the foot with the dynamometer would be more informative. Just to give the reader a clearer understanding of the testing setup.

Thank you for your comment.

Please see this change in the new Figure 2.

Statistics Section:

P9 Line 188 You analysed data in 1N blocks from 4N to 20N. Please can explain your reasoning (with references) for choosing this level of force. This is a relatively low level of force compared to other studies you have quoted such as Wilken et al (2011) who applied 45 to 111N (5 to 25Nm) of force.

Thank you for this relevant comment. We decided to analyzed data between 4 and 20N according to Barouk et al. recommendation [5]. They recommended a force between 1.7 and 2 kg. We daily use this amount of pressure at our clinic to diagnose gastrocnemius tightness in patients. We believe that increasing the force might decrease the reliability: it is important to control the patient's muscle relaxation (no muscle contraction), the hindfoot alignment... To do all this while trying to apply an important force could be challenging. Moreover, in painful patient it may be difficult to increase the force. Our study focused on the reliability of the device. Increasing the pressure would increase ankle dorsiflexion but would not change calf tightness. The first step is to develop a reliable device, which we did, and the second step will be to define the angular threshold of dorsiflexion by comparing pathological and control patients. Increasing the force would only change the angular threshold but not the tightness.

We added a sentence on that in our limitation section: 

P22- L391: “We only analyzed data between 4 and 20N, whereas some authors propose higher forces [10–12]. However, we designed our device according to Barouk et al. recommendations [5] in order to be as close as possible to the forces used during patients examination by physicians when performing a Silfverskiöld test”.

P9 Line 192 Please change ET to SD in the formula used for SEM, as you use SD in the rest of the document.

Thanks for the remark. We have made this correction. 

P10 L217: SEM = SD × (1-r)1/2

P9 Line 198 Please can you state which form of ICC you used i.e. ICC (3,1)

For this part, we referred to the article by Wilken et al. [12]. we have modified the explanation of the statistics part as follows:

P10-L222: “The intra-rater reliability was measured with Intraclass Correlation Coefficients (ICC) both for single measures, both comparing the three trials and taking the average of the three trials. The intra-rater reliability for single measures (SM) was calculated using a two-way random, single score ICC (ICC2,1) whereas the intra-rater reliability for average measures (AVG3M) was calculated using a two-way random, average score ICC (ICC2,3) [12].

We detailed which ICCs were used in each table, taking as reference the work of Wilken et al. [12]

Also, in the discussion you cite Koo et al (ref 25) for the level of reliability (good, excellent etc). I think it would be helpful to the readers if you put the range of values for each level of reliability as per Koo in this section.

Totally agree with your comment, it seems relevant to add the values according to Koo et al. to facilitate the reader's understanding

P12-L255: “The ICC values were between 0.84 and 0.99 (corresponding to a good and excellent reliability respectively, according Koo et al. [25]).”

P19 L313: “However, according to Koo et al. [25] (where values less than 0.5 are indicative of poor reliability, between 0.5 and 0.75 are moderate reliability, between 0.75 and 0.9 are good reliability, and greater than 0.90 are excellent reliability), …”

P9 Line 198 I am a bit confused by the single measures (SM) and average measures (AVG3M). I am not a statistics expert, but I suspect that if I am confused other readers will be too.

• Could you please describe why you are reporting single measures and average measures.

o I am guessing it is to show whether the test result is acceptable with just one measure (which makes it faster in clinic), or if performing it 3 times and using an average gives more accurate results. If that is the case, then you should make a comment in the results and discussion sections as to whether 1 or 3 trials is more acceptable and should be conducted when this device is used in the future.

As you point out in your review, the main reason for reporting both single measure ICCs and average measure ICCs is to assess whether it is worth performing 3 tests and taking the average among them or would it be more beneficial (and “cheap”) to use only one test. 

While most studies report ankle data (ROM) from an average of 3 tests, Wilken et al. [12] seem to suggest that one measure could be enough for certain instrument measures (IAROM).

In our study, we have considered both scenarios, and evaluated whether 3 tests are necessary or not. In view of our results, it finally seems that a single measurement is sufficient to obtain excellent reliability, which is of interest in clinical routine where the time available is a factor of choice for certain tools.

P20 L330: “Finally, like Wilken et al. [12], in view of our results, it seems that a single measurement is sufficient to obtain excellent reliability, rather than 3. This element is important to consider in order to develop a tool that can be easily used in clinical routine”

• Can you clarify what you mean by single measures (SM) and average measures (AVG3M).

o I assume the single measure (SM) is the reliability across each of the three trials for each participant for each rater?

o And that the AVG3M, is the average of the 3 trials for each participant for each rater? If this is the case then I am unsure what other value you are comparing this to, to calculate reliability, because you did not do a second set of three trials to produce another average.

Thank you. It is important to distinguish between interrater and intrarater reliability. 

For interrater reliability:

o The Single Measure ICC is an index for the reliability of each single rater

o The AVeraGe ICC is the average of the three trials for each rater

o The reliability is assessed by comparing the three raters

For the intrarater reliability:

o The Single Measure ICC is an index for the reliability of each trial

o The AVeraGe ICC is the average of the three raters for each trial

o The reliability is assessed by comparing the trials

See P10-11 L229

Results Section:

P10 Line 220 It would be useful to the reader if you stated the intra-rater results with the range of ICC and the level of reliability as per Koo et al.

Thank you for this remark. we added the following sentence, we hope this will meet your expectations: 

P11-L255: “The ICC values were between 0.84 and 0.99 (corresponding to a good and excellent reliability respectively, according Koo et al. [25]).”

P10 Line 220 It would be useful to the reader if you stated the inter-rater results with the range of ICC and the level of reliability as per Koo et al.

Thank again you for this remark. we added the following sentence. 

P12-L265: “The ICC where between 0.59 and 0.91 (corresponding to a moderate and excellent reliability respectively, according Koo et al. [25])”

P15 Table 4 As per my earlier comment regarding SM and AVG3M, I am not clear on what this is being reporting.

In the discussion you comment on the low SEM and MDC for inter rater testing. However, you have not reported SEM or MDC or mean ROM for intra tester reliability. I understand why you have not done this as the table with all 3 raters would be enormous. Nonetheless, it would be useful and important so readers can decide whether 1 or 3 trials are required for accurate results. Is it possible to include a table (similar format to table 3 inter rater results) of perhaps just one of the raters individual results with mean ROM, SEM and MDC to give an indication of the possible values. Or maybe it could be supplementary data.

You are right. We have therefore redone Table 2 in accordance with your comments, with SEM and MDC and data for each subject are in Supplementary Data. Thank you for this comment.

See Table 2 P13-14.

Discussion Section:

P18 Line 286 I agree with what you have written in this paragraph except for this sentence. The device is reliable for the levels of force applied but there has been no determination or evidence that your “optimal” force of 14-15N can identify gastrocnemius restriction.

Absolutely, we were not clear enough on this point. Our device has the best measurement reliability in this pushing interval (14-15N), but this does not predict that it will determine gastrocnemius tightness. This is a first step in our project.

P20-L323: “The equinometer described here sought to solve this problem by providing a standardized method with a reliable load application but the value presented as reliable in the present study does not constitute a threshold determining gastrocnemius tightness.”

P18, Line 288 This paragraph could do with more detail. Comparing and contrasting your results to other similar studies is important. But in this area the devices used are often quite different. So, a sentence on what device was used in the studies you have quoted, the amount of force applied and whether it was conducted weight bearing on non-weightbearing would be helpful.

Thank you. We have completed the discussion according to your comments:

P20-L327: “The intra-rater ICC obtained in this study was above 0.95, which is excellent reliability when compared to several studies of similar tools that were labelled as reliable [12,26–28]. However, it should be noted that for Larsen et al. [26] and Worsley et al. [28] the investigations were carried out in weight bearing conditions. Our inter-rater ICC values are not as good as those reported by Wilken et al. [12], who however used pushing intervals higher than the present study (between 10 N.m and 25 N.m) in non-weightbearing condition (ICC > 0.92).”

P21-L350: “Meyer et al. [29], who also used pushing intervals higher than the present study (10 N.m) in non-weightbearing condition also found differences in the ICC between the KE and KF conditions.”

P18, Paragraph from Line 311 Again the issue with calling 14 to 15N an “optimal” value. You have identified that it is optimal for producing the most reliable inter/intra rater results (and only within the limited range of forces you tested). You have not shown it to be optimal for identifying gastro tightness or any other pathology or indeed in what context that range is important. Consequently, I do not think you have supplied sufficient justification for recommending this range to be used for future testing.

Our study focused on the reliability of the device. Increasing the pressure would increase ankle dorsiflexion but would not change gastrocnemius tightness. The first step is to develop a reliable device, which we did, and the second step will be to define the angular threshold of dorsiflexion by comparing pathological and control patients. Increasing the force would only change the angular threshold but not the tightness. Now that we have a reliable device and force, further studies can be performed using these parameters to attempt to define the pathological threshold for gastrocnemius tightness.

We modified our manuscript according to your comment: 

P21- L360: “Determining the load application values to ensure the highest reliability was our study’s second objective.”

And P21-L365: “It is important to specify that the value presented as reliable in the present study does not constitute a threshold determining gastrocnemius tightness. Increasing the pressure would increase ankle dorsiflexion but would not change gastrocnemius tightness. The first step was to develop a reliable tool and the second step will be to define the angular threshold of dorsiflexion by comparing pathological and control patients. Once again, increasing the load would only change the angular threshold but not the tightness”

Conclusion

P20 Line 343 As per my previous comments, I do not think you can say the optimal level is appropriate for identifying gastro tightness.

We have modified the conclusion as follows:

P23-L403: “The equinometer was most reliable when pushing on the foot with a force between 14 and 15N. However, this does not inform the angular threshold for diagnosing gastrocnemius tightness. This validates a new equinometer as a reliable device. Further studies should be performed using these parameters to attempt to define the pathological threshold for gastrocnemius tightness.”

The authors

Attachment Submitted filename: Response to reviewers.docx Click here for additional data file.

# Peer review: Decision Letter 1

**DOI:** 10.1371/journal.pone.0284279.r003

**Authors:** Ravi Shankar Yerragonda Reddy

28 Mar 2023

Reliability of a new computerized equinometer based on Silfverskiöld test to measure gastrocnemius tightness

PONE-D-22-19979R1

Dear Dr. Lucas Martinez,

We’re pleased to inform you that your manuscript has been judged scientifically suitable for publication and will be formally accepted for publication once it meets all outstanding technical requirements.

Within one week, you’ll receive an e-mail detailing the required amendments. When these have been addressed, you’ll receive a formal acceptance letter and your manuscript will be scheduled for publication.

An invoice for payment will follow shortly after the formal acceptance. To ensure an efficient process, please log into Editorial Manager at http://www.editorialmanager.com/pone/, click the 'Update My Information' link at the top of the page, and double check that your user information is up-to-date. If you have any billing related questions, please contact our Author Billing department directly at authorbilling@plos.org.

If your institution or institutions have a press office, please notify them about your upcoming paper to help maximize its impact. If they’ll be preparing press materials, please inform our press team as soon as possible -- no later than 48 hours after receiving the formal acceptance. Your manuscript will remain under strict press embargo until 2 pm Eastern Time on the date of publication. For more information, please contact onepress@plos.org.

Kind regards,

Ravi Shankar Yerragonda Reddy, Ph.D

Academic Editor

PLOS ONE

Additional Editor Comments (optional):

The authors have addressed all the comments raised by the reviewers. The manuscript can be accepted in its current form.

Reviewers' comments:

Reviewer's Responses to Questions

**Comments to the Author**

1. If the authors have adequately addressed your comments raised in a previous round of review and you feel that this manuscript is now acceptable for publication, you may indicate that here to bypass the “Comments to the Author” section, enter your conflict of interest statement in the “Confidential to Editor” section, and submit your "Accept" recommendation.

Reviewer #1: All comments have been addressed

**********

2. Is the manuscript technically sound, and do the data support the conclusions?

The manuscript must describe a technically sound piece of scientific research with data that supports the conclusions. Experiments must have been conducted rigorously, with appropriate controls, replication, and sample sizes. The conclusions must be drawn appropriately based on the data presented. 

Reviewer #1: Yes

**********

3. Has the statistical analysis been performed appropriately and rigorously? 

Reviewer #1: Yes

**********

4. Have the authors made all data underlying the findings in their manuscript fully available?

The PLOS Data policy requires authors to make all data underlying the findings described in their manuscript fully available without restriction, with rare exception (please refer to the Data Availability Statement in the manuscript PDF file). The data should be provided as part of the manuscript or its supporting information, or deposited to a public repository. For example, in addition to summary statistics, the data points behind means, medians and variance measures should be available. If there are restrictions on publicly sharing data—e.g. participant privacy or use of data from a third party—those must be specified.

Reviewer #1: Yes

**********

5. Is the manuscript presented in an intelligible fashion and written in standard English?

PLOS ONE does not copyedit accepted manuscripts, so the language in submitted articles must be clear, correct, and unambiguous. Any typographical or grammatical errors should be corrected at revision, so please note any specific errors here.

Reviewer #1: Yes

**********

6. Review Comments to the Author

Please use the space provided to explain your answers to the questions above. You may also include additional comments for the author, including concerns about dual publication, research ethics, or publication ethics. (Please upload your review as an attachment if it exceeds 20,000 characters)

Reviewer #1: Thank you for responding and modifying all the comments from the previous review. Now the work can be accepted for publication.

Congratulations

**********

7. PLOS authors have the option to publish the peer review history of their article (what does this mean?). If published, this will include your full peer review and any attached files.

If you choose “no”, your identity will remain anonymous but your review may still be made public.

**Do you want your identity to be public for this peer review?** For information about this choice, including consent withdrawal, please see our Privacy Policy.

Reviewer #1: No

**********

<quillbot-extension-portal></quillbot-extension-portal>

# Editor report: Acceptance letter

**DOI:** 10.1371/journal.pone.0284279.r004

**Authors:** Ravi Shankar Yerragonda Reddy

31 Mar 2023

PONE-D-22-19979R1 

Reliability of a new computerized equinometer based on Silfverskiöld test to measure gastrocnemius tightness. 

Dear Dr. Martinez:

I'm pleased to inform you that your manuscript has been deemed suitable for publication in PLOS ONE. Congratulations! Your manuscript is now with our production department. 

If your institution or institutions have a press office, please let them know about your upcoming paper now to help maximize its impact. If they'll be preparing press materials, please inform our press team within the next 48 hours. Your manuscript will remain under strict press embargo until 2 pm Eastern Time on the date of publication. For more information please contact onepress@plos.org.

If we can help with anything else, please email us at plosone@plos.org. 

Thank you for submitting your work to PLOS ONE and supporting open access. 

Kind regards, 

PLOS ONE Editorial Office Staff

on behalf of

Dr. Ravi Shankar Yerragonda Reddy 

Academic Editor

PLOS ONE
